# Effects of Vibration Foam Rolling on Pain, Fatigue, and Range of Motion in Individuals with Muscle Fatigue: A Systematic Review

**DOI:** 10.3390/healthcare13121391

**Published:** 2025-06-11

**Authors:** Seju Park, Byeonggeun Kim

**Affiliations:** Department of Rehabilitation Health, Songwon University, Gwangju 61756, Republic of Korea

**Keywords:** vibration foam rolling, pain, muscle fatigue, range of motion, exercise, rehabilitation

## Abstract

**Background/Objectives:** Vibration foam rolling (VFR) has emerged as a popular intervention in sports and rehabilitation settings to enhance recovery and flexibility. This systematic review aimed to evaluate the effects of VFR on pain, fatigue, and range of motion (ROM) in individuals experiencing exercise-induced muscle fatigue and to assess its clinical applicability. **Methods**: A systematic literature search was conducted across five databases: PubMed, Cochrane Library, Embase, Web of Science, and CINAHL. Studies were included if they involved participants with muscle fatigue, applied VFR as an intervention, and measured outcomes related to pain, fatigue, or ROM. Methodological quality was assessed using the Joanna Briggs Institute critical appraisal tools. **Results**: Eight studies published between 2019 and 2024 met the inclusion criteria. VFR showed beneficial effects in reducing delayed onset muscle soreness, improving pressure pain threshold, and lowering subjective fatigue. Several studies also reported increased ROM in specific joints, including the hip and knee. However, findings across studies were inconsistent, particularly in physiological markers such as muscle oxygen saturation and blood flow parameters, where statistically significant differences were not always observed. **Conclusions**: VFR may offer potential benefits for pain relief, fatigue recovery, and ROM improvement in fatigued individuals. Nonetheless, its effects remain difficult to isolate from those of mechanical pressure and friction associated with foam rolling. Future studies with standardized intervention protocols and long-term follow-up are needed to clarify the independent role of vibration in recovery outcomes.

## 1. Introduction

Muscle fatigue is a physiological condition that arises after repetitive or high-intensity physical activity and is characterized by reduced muscle function and strength, the onset of pain, decreased physical performance, and limitations in range of motion (ROM) [1,2,3,4,5]. Not only healthy individuals and athletes but also patients are susceptible to muscle fatigue, with the latter being even more vulnerable due to underlying conditions often associated with impaired physical function [6,7]. Therefore, identifying effective interventions to alleviate muscle fatigue, facilitate recovery, and support post-exercise functional restoration has been considered essential in both sports and rehabilitation contexts.

Foam rollers have been widely used as recovery tools to relieve muscle fatigue, enhance flexibility, and promote post-exercise recovery [8,9]. Self-myofascial release using a foam roller has been reported to increase the elasticity of muscles and fascia, improve localized blood flow, and induce physiological recovery responses [10,11]. More recently, vibration foam rollers (VFRs), which integrate vibration with traditional foam rolling, have attracted attention as tools that offer greater improvements in ROM and physical performance compared to conventional foam rollers that rely solely on mechanical pressure [12,13]. Furthermore, VFRs have been shown to enhance recovery by improving blood flow parameters through vibratory stimulation [14]. VFR devices typically operate at frequencies ranging from 18 to 67 Hz [12,15,16,17]. A previous study reported that both low-frequency (35 Hz) and high-frequency (67 Hz) VFR interventions produced similar effects on knee flexion range of motion, muscle soreness, and jump performance. These findings suggest that the optimal frequency for specific recovery outcomes remains unclear and requires further investigation [16,17]. Owing to these various benefits, VFRs have been applied not only to healthy individuals and athletes but also to clinical populations, broadening their potential utility as rehabilitation tools [12,18,19].

Although VFR has been applied across diverse populations, it is primarily used to address exercise- or movement-induced muscle fatigue. Prior studies have explored its impact on various outcomes such as pain, ROM, and recovery markers. Some of these studies compared VFRs to non-vibration foam rollers (NVFRs), while others assessed VFRs alone. The findings, however, remain inconsistent. For instance, use of VFRs after eccentric exercise has shown greater short-term improvements in pain and passive hip extension ROM [20], whereas other studies reported no statistically significant advantages in terms of blood perfusion [21] or ROM, balance, and stability [22]. These mixed results highlight the need for a comprehensive synthesis of the evidence regarding the effectiveness of VFR in alleviating muscle fatigue, rather than determining its superiority over other interventions.

Accordingly, this systematic review aimed to examine the effects of VFR on pain, fatigue, and ROM in individuals with muscle fatigue. By synthesizing and analyzing previous studies on VFR interventions, this review aimed to provide clinical evidence and practical implications for its future application in sports and rehabilitation settings.

## 2. Materials and Methods

### 2.1. Study Design

This systematic review was conducted in accordance with the PRISMA (Preferred Reporting Items for Systematic Reviews and Meta-Analyses) guidelines to evaluate the effects of VFR on pain, fatigue, and ROM in individuals experiencing muscle fatigue [23]. Although we attempted to register the protocol with PROSPERO, the submission was not accepted. Therefore, in accordance with PRISMA 2020 checklist item 24b, we note that no protocol was ultimately registered.

The research question was defined using the PICOS framework, which includes population, intervention, comparison, outcomes, and study design. The population included individuals experiencing muscle fatigue induced by exercise or repetitive movements; the intervention involved the use of VFR; the comparison included NVFR or other recovery strategies; and the outcomes assessed were pain, fatigue, and ROM. The study designs included randomized controlled trial (RCT), as well as quasi-experimental designs, such as single-group pre–post studies and randomized or non-randomized crossover trials. Although some crossover trials employed randomization, we categorized them as quasi-experimental studies in this review.

### 2.2. Eligibility Criteria

Studies were included if they met the following criteria: (1) participants were healthy adults, athletes, or physically active individuals experiencing muscle fatigue induced by exercise (e.g., eccentric training, repetitive tasks, or sport-specific drills); (2) VFR was used as the primary intervention, applied independently or in comparison with other recovery tools; (3) pain, fatigue, or ROM were assessed as primary outcomes using validated instruments; (4) the study design was a randomized controlled trial or a quasi-experimental design, such as a single-group pre–post study or a crossover trial without randomization. Only peer-reviewed, full-text articles written in English were included. Studies were excluded if they were animal or in vitro studies, reviews or meta-analyses, conference abstracts, single-case reports, or if they lacked sufficient detail on intervention protocols, outcomes, or methodological transparency.

### 2.3. Search Strategy

The literature search was conducted on 7 September 2024, using the following databases: PubMed, Cochrane Library, Embase, Web of Science, and CINAHL. The search was performed without any restrictions on publication year. In PubMed, the representative search terms included “vibration foam roller” [TIAB] OR “vibration foam rolling” [TIAB] OR “vibration rolling” [TIAB] OR “vibration roller” [TIAB] OR “vibrating foam roller” [TIAB] OR “vibrating foam rolling” [TIAB] OR “vibrating roller” [TIAB] OR “vibrating rolling” [TIAB]. Although keywords related to the population were not included in the final search string, this decision was based on a preliminary search indicating that such additions significantly reduced the number of retrievable studies. To maximize sensitivity and ensure a comprehensive identification of studies on vibration foam rolling interventions, we adopted a broad search strategy focused on intervention-related terms.

### 2.4. Study Selection and Data Extraction

The titles and abstracts of the retrieved studies were screened to determine their eligibility, and full-text articles were subsequently reviewed to identify studies that met the inclusion criteria. From the final set of included studies, the following information was extracted: basic study characteristics (authors, year of publication, study design), participant characteristics (age, sex, and method used to induce muscle fatigue), intervention details (vibration intensity, application site, duration), and comparison group interventions (NVFR, no intervention, or other treatment methods). Outcome variables related to pain, fatigue, and ROM were extracted. In this review, pain-related outcomes included the Visual Analog Scale (VAS), muscle soreness, and pressure pain threshold (PPT). Fatigue-related outcomes encompassed both subjective assessments (e.g., perceived fatigue scores) and physiological markers such as blood lactate levels, creatine kinase, and skin blood flow. ROM-related outcomes covered measures of joint flexibility, such as range of motion or sit-and-reach performance. All procedures were independently performed by two reviewers, and any discrepancies were resolved through discussion.

### 2.5. Methodological Quality Assessment

The methodological quality of the included studies was assessed using the Joanna Briggs Institute (JBI) critical appraisal tools. Appropriate checklists were applied based on the study design; the 13-item JBI checklist was used for RCT, and the 9-item version was used for quasi-experimental studies [24,25]. The JBI appraisal tools assess the risk of bias, the validity of the study design, and the reliability of outcome reporting. Each item was rated as Yes (Y), Unclear (U), No (N), or Not Applicable (NA).

## 3. Results

### 3.1. Search Results

**Figure 1:**
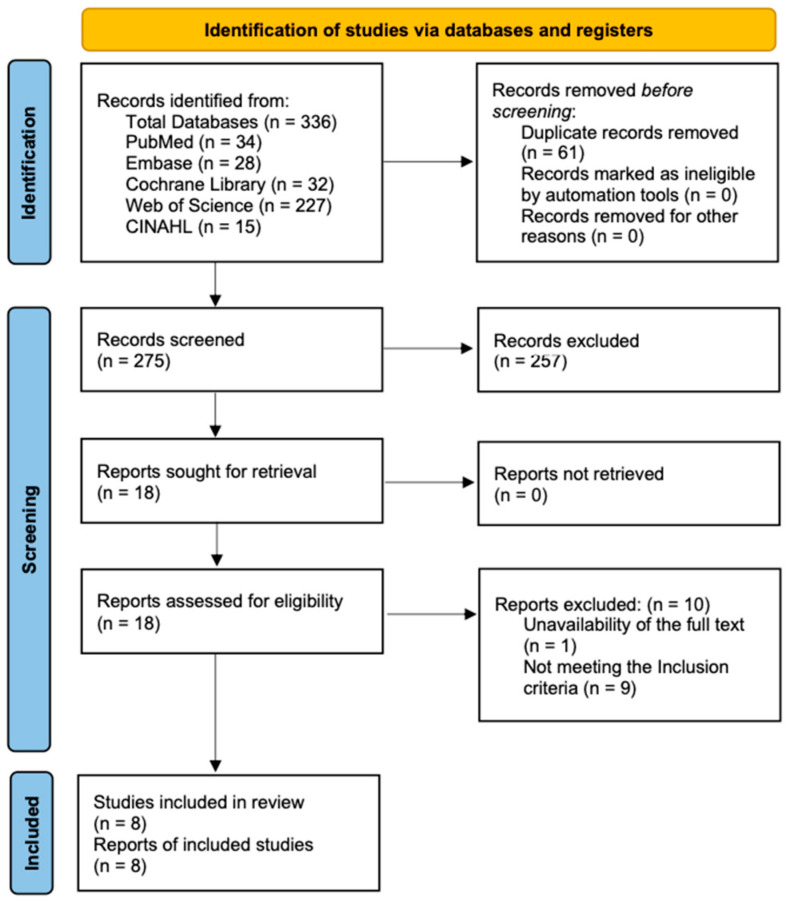
Flowchart describing the study selection process.

A total of 336 studies were identified through international database searches. The number of studies retrieved from each database was as follows: PubMed (*n* = 34), Cochrane Library (*n* = 32), Embase (*n* = 28), Web of Science (*n* = 227), and CINAHL (*n* = 15). After removing 61 duplicates, 275 studies remained. Titles and abstracts of these studies were screened, and 257 studies were excluded for not meeting the inclusion criteria, leaving 18 studies for full-text review. Following this, one study was excluded due to the unavailability of the full text, and nine additional studies were excluded for not meeting the inclusion criteria. As a result, a total of eight studies were included in this systematic review [20,21,22,26,27,28,29,30] (Figure 1).

### 3.2. Characteristics of Included Studies

A total of eight studies were included in this systematic review, all published between 2019 and 2024 (Table 1). Among them, three studies employed a RCT design, while five used a quasi-experimental design. The study populations consisted of athletes, healthy adults, and individuals in specialized professions involved in lifesaving activities. The methods used to induce muscle fatigue varied across studies and included sports training, eccentric contractions, and rescue-related physical activities. The intervention protocols also differed. VFR were applied with frequencies ranging from 18 Hz to 48 Hz. The targeted muscle groups included the quadriceps, hamstrings, and gastrocnemius. The intervention durations ranged from 30 s to 15 min, and most studies incorporated rest periods between sets. The comparison groups included NVFR, conventional recovery methods (e.g., stretching and walking), or no intervention. Primary outcome variables included pain (measured by VAS and PPT), fatigue (assessed via blood lactate levels, exercise-induced fatigue, creatine kinase levels, muscle oxygen saturation (SmO_2_), skin blood flow, vibratory blood flow analysis, and subjective fatigue ratings), and ROM (measured by ROM and sit-and-reach tests).

### 3.3. Methodological Quality

The methodological quality of the eight included studies was assessed using critical appraisal tools appropriate to their study designs (Table 2). Among the RCT, most items were rated as “Yes” [20,26,27]. However, some studies had items related to random sequence generation and allocation concealment rated as “Unclear” or “No.” For the quasi-experimental studies the overall methodologies were clearly described and showed high reliability [21,22,28,29,30]. Nevertheless, a few studies received “No” ratings for items concerning allocation procedures and the validity of the study design. Overall, all eight included studies met appropriate methodological standards for their respective designs, indicating a high level of reliability in their reported findings.

Randomized controlled trials (RCT): Q1. Was true randomization used for assignment of participants to treatment groups? Q2. Was allocation to treatment groups concealed? Q3. Were treatment groups similar at the baseline? Q4. Were participants blind to treatment assignment? Q5. Were those delivering the treatment blind to treatment assignment? Q6. Were treatment groups treated identically other than the intervention of interest? Q7. Were outcome assessors blind to treatment assignment? Q8. Were outcomes measured in the same way for treatment groups? Q9. Were outcomes measured in a reliable way? Q10. Was follow up complete and if not, were differences between groups in terms of their follow up adequately described and analyzed? Q11. Were participants analyzed in the groups to which they were randomized? Q12. Was appropriate statistical analysis used? Q13. Was the trial design appropriate and any deviations from the standard RCT design accounted for in the conduct and analysis of the trial? Quasi-Experimental studies: Q1. Is it clear in the study what is the “cause” and what is the “effect”? Q2. Was there a control group? Q3. Were participants included in any comparisons similar? Q4. Were the participants included in any comparisons receiving similar treatment/care, other than the exposure or intervention of interest? Q5. Were there multiple measurements of the outcome, both pre and post the intervention/exposure? Q6. Were the outcomes of participants included in any comparisons measured in the same way? Q7. Were outcomes measured in a reliable way? Q8. Was follow-up complete and if not, were differences between groups in terms of their follow-up adequately described and analyzed? Q9. Was appropriate statistical analysis used?

### 3.4. Effects on Pain

Among the eight studies included in this review, five examined the effects of VFR on pain reduction as a primary outcome. Romero-Moraleda et al. reported that VFR was more effective than NVFR in reducing delayed onset muscle soreness (DOMS) and improving pain sensitivity, with a 30.2% reduction in VAS scores (*p* = 0.033) [20]. PPT significantly increased in the vastus lateralis, rectus femoris, and vastus medialis, with the latter showing the most statistically significant improvement (*p* < 0.01). Similarly, Chang et al. demonstrated that pre-exercise application of VFR, used as a substitute for a general warm-up, significantly reduced VAS scores at 48 h (*p* = 0.006) and 72 h (*p* = 0.006) post-exercise in the quadriceps, and at 48 h in the hamstrings (*p* = 0.02) [27]. Nakamura et al. also reported that static compression VFR significantly decreased muscle soreness and increased PPT across multiple conditions, including maximal voluntary isometric contraction, maximal voluntary concentric contraction, stretching, and palpation (*p* < 0.01) [28]. Furthermore, Nakamura et al. found that applying VFR to the non-dominant limb significantly increased PPT and reduced pain on the affected side under maximal voluntary isometric contraction, maximal voluntary concentric contraction, and palpation conditions (*p* < 0.05) [29]. In contrast, Lai et al. observed lower VAS scores in the VFR group compared to the foam roller group (5.72 vs. 6.2), although the difference was not statistically significant (*p* = 0.327) [21]. Subjectively, 11 participants (48%) reported less pain with VFR, 7 (30%) reported less pain with FR, and 5 (22%) reported no difference. These findings suggest that VFR was perceived as more comfortable by a greater proportion of participants. Consistent with this, most studies reported positive effects of VFR on pain reduction, although some did not reach statistical significance.

### 3.5. Effects on Fatigue

Among the eight included studies, five reported outcomes related to fatigue. Zhu found that VFR significantly reduced post-exercise blood lactate levels (*p* < 0.05) and maintained lower subjective fatigue scores, indicating its effectiveness in promoting fatigue recovery (*p* < 0.05) [26]. Similarly, in the study by Chang et al., creatine kinase levels in the VFR group returned to baseline at 72 h post-exercise (*p* > 0.05), whereas the general warm-up group continued to show elevated creatine kinase levels at the same time point (*p* = 0.001), suggesting superior recovery of muscle damage in the VFR group [27]. Alonso-Calvete et al. also reported that VFR most effectively reduced blood lactate concentration (*p* = 0.001) and significantly lowered lower-limb fatigue, as measured by the rating of perceived exertion scale (*p* = 0.001), demonstrating strong recovery effects [30]. In contrast, Romero-Moraleda et al. observed that both the VFR and NVFR groups showed increases in SmO_2_ following exercise, but there was no significant difference between the groups (*p* > 0.05) [20]. Similarly, Lai et al. found that VFR increased skin blood flow, but no statistically significant difference was observed compared to the foam roller group (*p* = 0.894) [21]. In vibration-based blood flow analysis, increases were noted in endothelial (*p* = 0.690), neurogenic (*p* = 0.101), and myogenic (*p* = 0.401) activity, though none reached statistical significance. Taken together, several studies indicated that VFR had beneficial effects in reducing perceived fatigue and promoting physiological recovery, although some results were not statistically significant.

### 3.6. Effects on Range of Motion

Among the eight included studies, five evaluated the effects of VFR on ROM and flexibility. Romero-Moraleda et al. found that both VFR and NVFR increased ROM, with VFR producing a significantly greater improvement in hip extension ROM (*p* < 0.05) [20]. Similarly, Nakamura et al. reported that static VFR led to a 6.5% increase in knee flexion ROM (*p* < 0.01), indicating enhanced flexibility [28]. In another study by Nakamura et al., VFR was applied only to the non-damaged limb, and a significant increase in knee flexion ROM was observed on the opposite limb, suggesting a potential cross-education effect (*p* < 0.01) [29]. In contrast, Chang et al. observed no statistically significant changes in hip ROM following either VFR or general warm-up protocols (*p* = 0.43) [27]. Meanwhile, de Benito et al. found that both VFR and foam rolling significantly increased ankle ROM (*p* < 0.001) [22]. Although improvements were also noted in sit-and-reach flexibility, the difference was not statistically significant (*p* = 0.074) but was considered clinically meaningful. Overall, several studies reported that VFR had a positive impact on ROM and flexibility, though some did not show statistically significant differences.

## 4. Discussion

This systematic review aimed to evaluate the effects of VFR on pain, fatigue, and ROM in individuals with muscle fatigue. By synthesizing the findings of previous studies, this review sought to provide clinical evidence and practical implications for the application of VFR in exercise and rehabilitation settings. The results of this review indicate that VFR may have beneficial effects on pain reduction, fatigue recovery, and improvements in ROM. However, there was some variability among studies, and a few did not report statistically significant effects.

### 4.1. Effects on Pain

Several studies included in this review reported that VFR had positive effects on reducing DOMS and increasing PPT compared to NVFR. These effects were observed in studies applying VFR both as a pre-exercise intervention and as a post-exercise recovery tool [20,27,28,29]. While the underlying mechanisms may vary depending on the timing of application, proposed explanations include increased intramuscular blood flow and stimulation of sensory receptors.

These findings are consistent with previously proposed mechanisms of pain modulation by vibratory stimulation. According to the gate control theory, vibration activates large-diameter A-β sensory fibers through cutaneous receptors, which in turn stimulate inhibitory interneurons in the spinal cord. This process suppresses the activity of A-δ and C fibers, which are responsible for transmitting pain signals, thereby reducing the perception of pain [31,32,33]. This neurophysiological mechanism provides a theoretical basis for the analgesic effects of VFR. Although some studies reported greater pain reduction with VFR compared to NVFR, the statistical significance of these effects varied across studies [20,21,27,28,29]. These inconsistencies may be attributed to differences in vibration intensity, frequency, application duration, and the physical properties of the foam rollers used. However, few studies to date have directly compared these variables using standardized protocols. Therefore, to systematically evaluate the pain-relieving effects of VFR under varying conditions, future research should employ standardized experimental designs and conduct comprehensive meta-analyses.

### 4.2. Effects on Fatigue

The studies included in this review suggested that VFR may play a positive role in post-exercise fatigue recovery. Zhu and Alonso-Calvete et al. reported that VFR significantly reduced blood lactate concentrations at 30 min and 15 min post-exercise, respectively, and lowered subjective fatigue, as measured by the rating of perceived exertion [26,30]. Chang et al. found that creatine kinase levels in the VFR group returned to baseline more rapidly, indicating potential contributions to muscle damage recovery [27]. However, studies by Romero-Moraleda et al. and Lai et al. reported no statistically significant differences between the VFR and control groups in physiological markers such as SmO_2_, skin blood flow, endothelial activity, and neurogenic activity [20,21]. These mixed findings suggest that caution is warranted when interpreting the physiological effects of vibration.

While these results partially align with proposed physiological mechanisms underlying fatigue recovery, the lack of consistent significance across physiological variables raises the possibility that the primary effects may stem from mechanical stimulation namely, the pressure and friction applied by the foam roller rather than vibration itself. Foam rolling has been reported to promote vasodilation, facilitate oxygen delivery, and enhance mitochondrial metabolism, thereby supporting the resynthesis of adenosine triphosphate and phosphocreatine [20,34]. Based on current evidence, it remains difficult to conclude that vibration alone enhances the recovery effects of foam rolling. Future studies should standardize variables such as pressure intensity, application duration, and target site, in addition to comparing vibration and non-vibration conditions, to more clearly isolate the independent effects of vibration stimuli.

### 4.3. Effects on Range of Motion

Improvements in ROM were observed in studies by Nakamura et al. and Romero-Moraleda et al., which reported increased hip and knee flexion ROM following VFR interventions [20,28,29]. Notably, Nakamura et al. demonstrated a cross-education effect, where VFR applied to the non-dominant limb led to improved knee flexion ROM on the contralateral, affected side [29]. In contrast, studies by Chang et al. and de Benito et al. did not report statistically significant improvements in ROM or flexibility, making it difficult to draw consistent conclusions regarding the effectiveness of VFR in enhancing ROM [22,27].

These findings partially align with physiological theories suggesting that vibration stimuli may enhance joint mobility by altering the thixotropic properties of muscle and fascial tissues. Vibration may also influence the proprioceptive system by temporarily reducing muscle tone and expanding movement range through pain relief. The cross-education effect observed by Nakamura et al. points to potential neurophysiological mechanisms, such as central nervous system modulation and improved proprioceptive feedback [29]. However, the inconsistency in statistical significance and effect sizes across studies limits the ability to confirm the effectiveness of VFR on ROM. In some cases, VFR showed effects similar to those of standard foam rollers, despite the addition of vibration. These inconsistencies are in line with findings from a previous meta-analysis, which also noted considerable heterogeneity among studies on this topic [12]. It is possible that mechanical factors such as pressure, friction, and localized heat generated by foam rolling may play a more significant role than vibration itself in improving ROM. Therefore, future studies should not only compare vibration and non-vibration conditions but also systematically control for vibration parameters such as application duration, intensity, and frequency to clarify the mechanisms behind ROM improvement.

### 4.4. Clinical Implications

The findings of this systematic review suggest that VFR may positively influence pain relief, subjective fatigue recovery, and improvements in ROM in individuals experiencing muscle fatigue. These potential benefits indicate that VFR could be clinically applicable in various populations where recovery and flexibility are particularly important, such as athletes, patients undergoing rehabilitation, and older adults. Given its non-invasive and relatively simple nature, VFR may serve as a convenient adjunct to pre- and post-exercise recovery routines or as a supplementary tool in rehabilitation programs. However, the effects of vibratory stimulation could not be clearly distinguished from the mechanical stimuli inherent in foam rolling, such as pressure, friction, and tissue mobilization. Moreover, the physiological markers assessed across studies did not consistently demonstrate statistically significant effects attributable solely to vibration. As such, there is currently insufficient clinical evidence to mandate the inclusion of vibration in foam rolling interventions. Instead, a personalized approach that considers user response, target application areas, and levels of fatigue is recommended. Additionally, standardized guidelines regarding vibration intensity, frequency, and duration are lacking, highlighting the need for more refined clinical application strategies in future research and practice.

### 4.5. Limitations and Future Directions

This systematic review has several limitations. It included a limited number of studies, and there was considerable heterogeneity among them in terms of intervention protocols, vibration characteristics, and assessment methods. Additionally, most studies involved small sample sizes, and the proportion of RCT was insufficient. These factors necessitate caution in interpreting the results and limit the generalizability of the findings. Future research should include large-scale RCT that control for various types of exercise and muscle fatigue induction protocols. Moreover, further studies are needed to identify optimal VFR intervention parameters, including frequency, intensity, application site, and duration. Long-term investigations are also warranted to assess the impact of VFR on functional recovery, injury prevention, and athletic performance enhancement. Furthermore, it is noteworthy that all studies included in this review focused exclusively on the application of VFR to the lower extremities, likely due to the frequent use of leg muscles in fatigue protocols, the ease of self-application, and the early stage of research in this area.

## 5. Conclusions

This systematic review examined the effects of VFR on pain relief, fatigue recovery, and improvements in ROM in individuals with exercise-induced muscle fatigue, and assessed its clinical applicability. The included studies reported some beneficial effects of VFR, particularly in reducing subjective fatigue, alleviating DOMS, and enhancing ROM in specific joints. However, the consistency of results across studies was limited, and statistically significant differences in physiological markers were not consistently observed. While these findings partially support the physiological mechanisms suggesting that vibration may aid in recovery, they also highlight a limitation in clearly distinguishing the effects of vibration from the mechanical stimuli inherent in foam rolling, such as pressure and friction. To more clearly validate the independent effects of vibratory stimulation, future studies should incorporate control groups with and without vibration, standardize intervention parameters, and investigate the long-term effects of VFR across various populations and settings.

## Figures and Tables

**Table 1:** Characteristics of studies included in the systematic review.

Author (Year)	Study Design	Sample	Fatigue Protocol	VFR Intervention (Freq, Site, Duration)	Comparison Group	Outcome Measures
Romero-Moraleda et al., 2019 [20]	RCT	*n* = 38 healthy adults (19 VFR, 19 NVFR), VFR: 21.9(3.7) y, 1.77(0.07) m, 75.3(8.0) kg; NVFR: 22.2(3.2) y, 1.74(0.07) m, 69.7(11.4) kg	Eccentric squats	18 Hz, Bilateral quadriceps, 5 min per leg, applied 48 h post-exercise	NVFR	VAS↓ (−30.2%, *p* = 0.033); PPT↑ (notably vastus medialis, *p* < 0.01); SmO_2_↑ (no between-group difference); ROM↑ (VFR > NVFR in hip extension, *p* < 0.05)
Lai et al., 2020 [21]	Quasi -experimental	*n* = 23 healthy adults (Male: 26.2(5.2) y, 169.5(3.9) cm; Female: 26.6(7.8) y, 156.8(5.1) cm)	50 min treadmill	20–40 Hz (mixed), Bilateral gastrocnemius, 3 min per	NVFR	VAS↓ in VFR (5.72 vs. 6.2, *p* = 0.327; 48% preferred VFR); SBF↑ in VFR (NS vs. FR); BFO↑ (endothelial, neurogenic, myogenic components all not significant)
de Benito et al., 2019 [22]	Quasi -experimental	*n* = 24 Male (*n* = 17): 22.5 y, 177.03 cm, 73.43 kg Female (*n* = 7): 20.43 y, 160.86 cm, 56.64 kg	Repetitive lunges at 30 reps/min until volitional fatigue	30 Hz, bilateral quadriceps and hamstrings, 2 × 60 s per muscle with 30 s rest	NVFR, No treatment	Ankle ROM↑ in both groups (*p* < 0.001); flexibility (sit-and-reach)↑ (*p* = 0.074, clinically meaningful)
Zhu, 2023 [26]	RCT	36 male college basketball players (19.8–20.4 y); EXP *n* = 18, CON *n* = 18	Simulated game drills	Main muscle groups, 15 min per session, 3 times per week, for 9 weeks	Walking and stretching for 15 min	Lactic acid↓ (*p* < 0.05); subjective fatigue↓ (*p* < 0.05), supporting fatigue recovery
Chang et al., 2024 [27]	RCT	VFR group and General Warm-up group, 24 male college athletes (handball and rugby players). 20.21(1.15) y, 175.92(5.57) cm, 76.25(9.13) kg	15 sets of maximal multidirectional repeated sprints	48 Hz, Quadriceps and hamstrings, 30 s/set at 30 rolls/min, 4 sets, Total 8 min	General Warm-up group	VAS↓ at 48/72 h post-ex (quad, ham; *p* < 0.01); CK returned to baseline at 72 h in VFR (vs. elevated in control, *p* = 0.001); hip ROM: no significant change (*p* = 0.43)
Nakamura et al., 2022 [28]	Quasi -experimental	*n* = 14 healthy young male, 20.4(0.8) y, 170.9(6.8) cm, 65.1(9.3) kg	Eccentric knee extensions	35 Hz, Midpoint of the dominant-side quadriceps, 3 × 30 s with 30 s rest, Total 90 s	-	Muscle soreness↓ (*p* < 0.01); PPT↑ across multiple conditions (*p* < 0.01); knee flexion ROM↑ by 6.5% (*p* < 0.01)
Nakamura et al., 2022 [29]	Quasi -experimental	*n* = 14 healthy young male, 21.4(0.7) y, 171.0(5.8) cm, 65.3(8.2) kg	Eccentric contraction of the dominant leg knee extensors	35 Hz, Quadriceps of the nondamaged leg, 3 × 30 s with 30 s rest, Total 90 s	-	Pain↓ and PPT↑ on affected side (*p* < 0.05) despite VFR applied to opposite limb; knee flexion ROM↑ on contralateral side (*p* < 0.01), suggesting cross-education effect
Alonso-Calvete et al., 2021 [30]	Quasi -experimental	*n* = 7 certified lifeguards (2 Female, 5 Male), mean age 23.29(1.11) y, 173.43(8.6) cm, 76.57(5.94) kg	Simulated 100 m water rescue (swimming with fins, towing manikin, extracting to shore)	18 Hz, quadriceps and hamstrings (both legs), 2 × 30 s per muscle with 15 s rest	Passive recovery (seated), non-vibrating foam roller	Lactate↓ (*p* = 0.001); perceived lower-limb fatigue↓ (RPE, *p* = 0.001)

RCT—randomized controlled trial; VFR—vibration foam roller; NVFR—non-vibration foam roller; VAS—visual analog scale; PPT—pressure pain threshold; SmO_2_—muscle oxygen saturation; ROM—range of motion; SBF—skin blood flow; BFO—blood flow oscillation; CK—creatine kinase; RPE—rated perceived exertion scale.

**Table 2:** Methodological quality assessment with JBI.

Author (Year)	Q1	Q2	Q3	Q4	Q5	Q6	Q7	Q8	Q9	Q10	Q11	Q12	Q13
Romero-Moraleda et al., 2019 [20]	Y	U	Y	N	N	Y	Y	Y	Y	Y	Y	Y	Y
Lai et al., 2020 [21]	Y	N	Y	Y	Y	Y	Y	Y	Y	-	-	-	-
de Benito et al., 2019 [22]	Y	Y	Y	Y	Y	Y	Y	Y	Y	-	-	-	-
Zhu, 2023 [26]	Y	U	Y	N	N	U	Y	Y	Y	Y	Y	Y	Y
Chang et al., 2024 [27]	Y	U	Y	N	N	U	Y	Y	Y	Y	Y	Y	Y
Nakamura et al., 2022 [28]	Y	N	Y	Y	Y	Y	Y	Y	Y	-	-	-	-
Nakamura et al., 2022 [29]	Y	N	Y	Y	Y	Y	Y	Y	Y	-	-	-	-
Alonso-Calvete et al., 2021 [30]	Y	Y	Y	Y	Y	Y	Y	Y	Y	-	-	-	-

## Data Availability

Data can be requested from the corresponding author and will be released on reasonable request.

## Abbreviations

The following abbreviations are used in this manuscript:

ROM	Range of motion
VFR	Vibration foam roller
NVFR	Non-vibration foam roller
RCT	Randomized controlled trial
JBI	Joanna Briggs Institute
VAS	Visual analog scale
PPT	Pressure pain threshold
SmO_2_	Muscle oxygen saturation
DOMS	Delayed onset muscle soreness
